# Correction to ‘Organic residue analysis shows sub-regional patterns in the use of pottery by Northern European hunter–gatherers’

**DOI:** 10.1098/rsos.201168

**Published:** 2020-07-22

**Authors:** Blandine Courel, Harry K. Robson, Alexandre Lucquin, Ekaterina Dolbunova, Ester Oras, Kamil Adamczak, Søren H. Andersen, Peter Moe Astrup, Maxim Charniauski, Agnieszka Czekaj-Zastawny, Igor Ezepenko, Sönke Hartz, Jacek Kabaciński, Jacek Kabaciński, Andreas Kotula, Stanisław Kukawka, Ilze Loze, Andrey Mazurkevich, Henny Piezonka, Gytis Piličiauskas, Søren A. Sørensen, Helen M. Talbot, Aleh Tkachou, Maryia Tkachova, Adam Wawrusiewicz, John Meadows, Carl P. Heron, Oliver E. Craig

*R. Soc. Open Sci.* **7**, 192016. (Published Online 22 April 2020) (doi:10.1098/rsos.192016)

The purpose of this correction is to update the Acknowledgements section with the below text. The original paper has been corrected.

We would like to express our gratitude to all of the institutions and museums who kindly provided access to their pottery collections and permissions to sample the vessels analysed in this study: the Institute of Archaeology and Ethnology of the Polish Academy of Sciences, the Institute of History of the National Academy of Sciences of Belarus, Moesgård Museum, Museum Lolland-Falster, Muzeum Podlaskie w Białymstoku, Nalsia Museum, the National History Museum of Latvia, the National Museum of Lithuania, Nicolaus Copernicus University, the Schleswig-Holstein State Museums Foundation Schloss Gottorf, The State Hermitage Museum, Tallinn University Archaeological Research Collection, and the University of Tartu archaeology collections.

