# Supplementary material for: Organic residue analysis shows sub-regional patterns in the use of pottery by Northern European hunter–gatherers
Source: R Soc Open Sci. 2020 Apr 22;7(4):192016. doi: 10.1098/rsos.192016 (PMC7211838; doi:10.1098/rsos.192016)
Supplement: Supplementary Information [file rsos192016supp2.docx]

**Supplementary Information**

**Organic residue analysis shows sub-regional patterns in the use of pottery by Northern European hunter-gatherers**

Blandine Courel^a,1,2^, Harry K. Robson^b,1^, Alexandre Lucquin^b,1^, Ekaterina Dolbunova^a,c^, Ester Oras^d^, Kamil Adamczak^e^, Søren H. Andersen^f^, Peter Moe Astrup^f^, Maxim Charniauski^g^, Agnieszka Czekaj-Zastawny^h^, Igor Ezepenko^g^, Sönke Hartz^i^, Jacek Kabaciński^j^, Andreas Kotula^k^, Stanislaw Kukawka^e^, Ilze Loze^l^, Andrey Mazurkevich^c^, Henny Piezonka^m^, Gytis Piličiauskas^n^, Søren A. Sørensen^o^, Helen M. Talbot^b^, Aleh Tkachou^g^, Maryia Tkachova^g^, Adam Wawrusiewicz^p^, John Meadows^q^, Carl P. Heron^a^ and Oliver E. Craig^b,2^

^a^Department of Scientific Research, The British Museum, London, WC1B 3DG, UK

^b^BioArCh, Department of Archaeology, University of York, York, YO10 5DD, UK

^c^The State Hermitage Museum, 34 Dvortsovaya Embankment, Saint Petersburg 190000, Russian Federation

^d^Institute of Chemistry, University of Tartu, Ravila 14A, 50411, Tartu, Estonia

^e^Institute of Archaeology, Nicolaus Copernicus University, Szosa Bydgoska 44/48, 87-100 Toruń, Poland

^f^Moesgård Museum, Moesgård Alle 15, Højbjerg, 8270 Denmark

^g^Department of Archaeology of Prehistoric Society, Institute of History, National Academy of Sciences of Belarus, Academic St 1, 220072 Minsk, Belarus

^h^Institute of Archaeology and Ethnology, Polish Academy of Science, Sławkowska 17, 31-016 Krakow, Poland

^i^Stiftung Schleswig-Holsteinische Landesmuseen, Schloss, Gottorf, D-24837 Schleswig, Germany

^j^Institute of Archaeology and Ethnology Polish Academy of Science, 31-016 Kraków, Poland

^k^Seminar für Ur- und Frühgeschichte, Georg-August-Universität Göttingen, Nikolausberger Weg 15, 37073 Göttingen

^l^Institute of Latvian History, University of Latvia, Rīga LV-1050, Latvia

^m^Institut für Ur- und Frühgeschichte, Christian-Albrechts-Universität zu Kiel, Johanna-Mestorf-Straße 2-6, D - 24118 Kiel, Germany

^n^Lithuanian Institute of History, Kražių st. 5, Vilnius 01108, Lithuania

^o^Museum Lolland-Falster, Frisegade 40, 4800 Nyk. F., Denmark

^p^Muzeum Podlaskie w Białymstoku, Ratusz, Rynek Kościuszki 10, 15 - 426 Białystok, Poland

^q^Centre for Baltic and Scandinavian Archaeology (ZBSA), Schleswig-Holstein State Museums Foundation, Schloss Gottorf, Schlossinsel 1, D-24837 Schleswig, Germany

^1^These authors contributed equally to this work.

^2^To whom correspondence may be addressed. Email: [bcourel@britishmuseum.org](mailto:bcourel@britishmuseum.org) or [oliver.craig@york.ac.uk](mailto:oliver.craig@york.ac.uk)

**Supplementary Materials and Methods**

**Modern reference material**

Forty-two modern animal tissue samples were extracted and analyzed according to the same protocols as the pottery material using the direct *in situ* transesterification extraction method. A dataset (Dataset 2) was compiled for the Western and Eastern Baltic. This included carbon (δ^13^C) isotope values of *n*-hexadecanoic (C_16:0_) and *n*-octadecanoic (C_18:0_) acids from authentic reference animal tissues and included new as well as published data [[1–8]](https://paperpile.com/c/eHRMPd/IHeKW+H7sBG+tWjYf+GONNg+G64D+qycmX+tuRk5+lVgUu). To facilitate comparison with the archaeological data, the values were adjusted given the variation in the atmospheric δ^13^C resulting from post-industrial carbon [[9]](https://paperpile.com/c/eHRMPd/Sxa9i) according to the known or estimated year of death of the animal. The post-industrial carbon contribution in modern marine animal was estimated at 40% in the Western Baltic and 90% in the Eastern Baltic [[10]](https://paperpile.com/c/eHRMPd/nZ7xX).

**Bayesian mixing model**

Modelling was carried out using the 3.0 Beta version (<https://sourceforge.net/projects/fruits/>) of the Bayesian mixing model FRUITS [[11]](https://paperpile.com/c/eHRMPd/bFjdh). Firstly, a model was implemented using the δ^13^C_16:0_ and δ^13^C_18:0_ values as proxies, without priors. Four food groups were selected as potential sources: marine, freshwater, porcine and ruminant, combining dairy and adipose reference material (Table S2). Carbon isotope values were obtained from the modern reference material dataset (Dataset 2) using specific source values for the western and Eastern Baltic. Uncertainties were derived using a covariance matrix and standard errors of the mean δ^13^C values for each food source, assuming a multivariate normal distribution and that the vessels were used repeatedly. Palmitic and stearic acid concentration values were based on the USDA Food Composition Databases (<https://fdc.nal.usda.gov/>; USDA National Nutrient Database for Standard Reference 28 Software v.3.8.6.1, downloaded on the 4^th^ of September 2017; Table S3; [[12]](https://paperpile.com/c/eHRMPd/9zZd)) with uncertainties expressed by their standard deviations. The concentration parameters are expressed as percentage of total lipids. The concentrations and model outputs are expressed as percentage of total lipid by weight. Numerical Bayesian inference was performed using the BUGS software, a Markov chain Monte Carlo (MCMC) method that employs Gibbs sampling and the Metropolis-Hastings algorithm. The first 5,000 iterations of the MCMC chains were discarded (burn-in steps) and these were then run for an additional 10,000 iterations. Model convergence was checked by inspecting if the trace plots of the respective posterior chains exhibited an asymptotic behaviour. Trace autocorrelation plots were also inspected to assess convergence.

A second model using the same parameters as above increased the model to five sources (ruminant adipose, ruminant dairy, porcine, marine and freshwater) (Table S2) and was applied to 15 samples given their large isotopic discrepancy between the main fatty acids characteristic of ruminants (Δ^13^C < -4.3‰).

**Supplementary Results and Discussion**

**Comments concerning the vessels containing dairy products**

Four of the vessels for which the mixing model showed a possible contribution of dairy products were securely assigned to the Late Mesolithic Ertebølle culture. The vessels were derived from the sites of Neustadt and Grube-Rosenhof in Northern Germany. Although these sites have younger Early Neolithic layers with domesticated fauna dating from *ca*. 4000 cal BC [[13]](https://paperpile.com/c/eHRMPd/M1WTp), these layers are clearly associated with distinctive Funnel Beakers (TRB) that vary considerably in form and manufacture from the Ertebølle vessels analyzed. The Ertebølle sherds were represented by thick-walled pointed-based vessels of various sizes and all were made using the U-technique. The two vessels from Neustadt, N1191i and N1910i, were represented by a body/rim sherd and rim sherd respectively (Fig. S6) and are typologically dated from *ca*. 4600-4000 cal BC. Moreover, the two vessels from Grube-Rosenhof, ROS 847-I and ROS 870-F, were represented by a rim sherd and base sherd respectively (Fig. S6). Of these, one (ROS 870-F) has been directly dated to 4550-4450 cal BC (SUERC-89944, 5658 ± 23 BP; Fig. S3).

In contrast, the vessel from Kaldus (site 3) in Northern Poland was assigned to the Narva culture. It was made using the N-technique and had shell temper as well as net-ornamentation on its exterior, which had probably been produced using a comb-tool; all of which are uncharacteristic features of early pottery from the Lower Vistula region. The vessel, POL-267, was represented by a rim sherd (Fig. S6). Interestingly, another sampled sherd from the site, POL-266, showed a strong resemblance with Narva pottery found at Zvidze (suggesting some relationship between the two sites). Since neither vessels have been directly dated, they may belong to a younger phase of the Narva culture given the broad range for this final sequence [[14]](https://paperpile.com/c/eHRMPd/Ecdba).

**AMS radiocarbon (^14^C) dating**

We have not critically reassessed the absolute chronologies of all the assemblages sampled in this study, but a small number of previously unpublished AMS dates are now available, which support the general chronological placement of the various pottery types discussed in the main text. These results (Fig. S3) were obtained at the Leibniz Laboratory, Christian-Albrechts-University Kiel (KIA-), the Scottish Universities Environmental Research Centre, East Kilbride (SUERC-) or the Oxford Radiocarbon Accelerator Unit, Oxford (OxA-). Samples were extracted following routing chemical pre-treatment methods, described in [[15]](https://paperpile.com/c/eHRMPd/vBNRk), [[16]](https://paperpile.com/c/eHRMPd/eRHgl) and [[17]](https://paperpile.com/c/eHRMPd/oStWo).

**Comments regarding the ^14^C dating results**

*Grube-Rosenhof (site 7, Fig. S1)*

One interior foodcrust adhering to an Ertebølle vessel (ROS 870-F) was sampled for radiocarbon dating. The molecular and isotopic analysis demonstrated that the lipid profile was terrestrial in nature (i.e. Δ^13^C value, *SRR*% and the absence of APAA C_20_ and C_22_), and consistent with dairy products (Bayesian model estimate, see Table S5). The ^14^C result (SUERC-89944, 5658 ± 23 BP) provides the earliest direct date for dairy products in the Western Baltic. Simple calibration gives a 95% probability range of 4550-4450 cal BC (Fig. S3), which might be regarded as a maximum age, given the possibility that some of the carbon extracted from the foodcrust was of marine origin. Even if a significant proportion of the carbon in the foodcrust came from marine sources, however, this vessel must predate the appearance of farmers with Funnel Beaker pottery in this area at *ca*. 4000 cal BC. The result therefore implies commodity exchange between Ertebølle hunter-gatherers at Rosenhof and, for example, Rössen farmers further south. That being accepted, there is no reason to assume that the simple calibrated date is misleadingly early.

*Lučyn Barok Siamionaŭski (site 33, Fig. S1)*

This sample lacked aquatic biomarkers and had a low *SRR*% (60%), consistent with ruminant carcass fats. Furthermore, the measured δ^13^C values of the two main fatty acids (C_16:0_ and C_18:0_) were relatively high, indicative of terrestrial ingredients. Thus, while the calibrated foodcrust ^14^C age should be interpreted as a maximum age for the pot, any freshwater reservoir effect is likely to have been minimal. In the absence of dates from fully terrestrial materials associated with this assemblage, or from other assemblages of this pottery type, even a maximum age is significant.

*Riigiküla VI (site 54, Fig. S1)*

Two new dates on calcined mammal bone fragments from the cultural layer with Narva pottery are marginally later than the only published date from this site (Hela-1909, 6130 ± 45 BP; [[18]](https://paperpile.com/c/eHRMPd/Tg16l)), on another calcined bone fragment. Dating to the early 5^th^ millennium, these samples are nevertheless relatively early for Estonian Narva Ware sites [[14]](https://paperpile.com/c/eHRMPd/Ecdba).

*Zvidze (site 62, Fig. S1)*

The Mesolithic-Early Neolithic sequence at Zvidze was originally dated by a series of radiometric ^14^C dates from the Tartu laboratory on waterlogged wood, and additional dates on bulk peat from a number of radiometric laboratories in the former Soviet Union [[19]](https://paperpile.com/c/eHRMPd/pmhzf). Depending on which of the wood samples were associated with pottery, these results date the appearance of Early Narva Ware to *ca*. 5500 cal BC. One sherd analysed in this study, from Layer 13 (the deepest layer with pottery) had a charcoal fragment embedded in the surface of the pottery fabric, which was dated to 5620-5590 cal BC (7%) or 5570-5470 cal BC (88% probability) (KIA-53101, 6567 ± 33 BP). The charcoal was identified by Dr Henrike Effenberger as alder (*Alnus* sp.) wood that was >10 cm in diameter. Its core and bark were absent. This means that an intrinsic age of several decades is likely (i.e. the calibrated date of this sample is probably several decades earlier than the tree-fall date), but it seems unlikely that there was a significant time lag between tree-fall and the production of the pot. The new date therefore seems to confirm that Narva-style pottery appeared in South-eastern Latvia by the mid-6^th^ millennium cal BC.

**Supplementary Information: Figures**

**
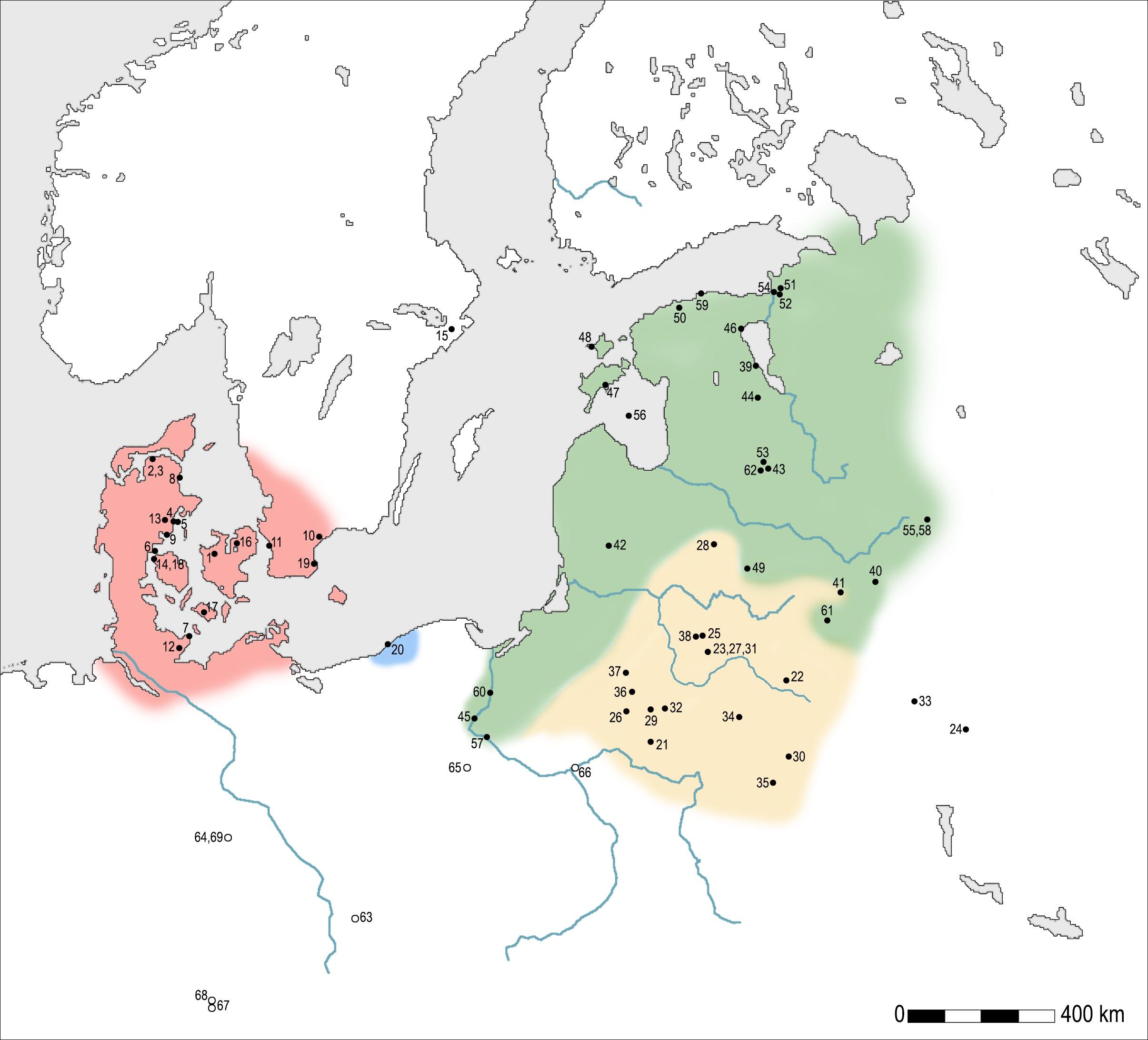
**

**Fig. S1**. Site locations and early ceramic-producing cultural groups of hunter-gatherers during the late 6^th^ and 5^th^ millennium cal BC in which pottery was sampled in this study. For comparison, contemporary agricultural LBK sites are also marked. 1: Åkonge, 2: Åle, 3: Bjørnsholm, 4: Flynderhage, 5: Frederiksodde, 6: Gamborg Fjord, 7: Grube-Rosenhof LA 58, 8: Havnø, 9: Hjarnø, 10: Kesemölla, 11: Löddesborg, 12: Neustadt LA 156, 13: Ringkloster, 14: Ronæs Skov, 15: Soldattorpet, 16: Stenø, 17: Syltholm, 18: Tybrind Vig, 19: Vik, 20: Dąbki, site 9, 21: Bransk, site 22, 22: Drazdy 12, 23: Dubičiai 3, 24: Dubovy Loh 5, 25: Glūkas 3, 26: Grądy-Woniecko, site 1, 27: Gribaša 4, 28: Jara 2, 29: Jeroniki, site 2, 30: Kamen’ 6, 31: Karaviškės 6, 32: Krzemienne, site 2, 33: Lučyn Barok Siamionaŭski, 34: Rusakova, 35: Sien’čycy 3, 36: Sośnia, site 1, 37: Stacze, site 1, 38: Varėnė 10, 39: Akali, 40: Asaviec 4, 41: Biarešča 4, 42: Daktariškė 5, 43: Iča, 44: Kääpa, 45: Kaldus, site 3, 46: Kalmaküla, 47: Kõnnu, 48: Kõpu 1, 49: Kretuonas 1C, 50: Kroodi, 51: Lommi III, 52: Narva Joaorg, 53: Osa, 54: Riigiküla IV, VI, 55: Rudnya Serteyskaya, 56: Ruhnu II, 57: Sasieczno, site 4, 58: Serteya X, XIV, 59: Vihasoo III, 60: Welcz Wielki, site 10A, 61: Zacennie, 62: Zvidze. LBK farmer sites nearby 63: Bylany, 64: Eythra, 65: Kopydłowo, site 6, 66: Ludwinowo site 7, 67: Niederhummel, 68: Wang, 69: Zwenkau.


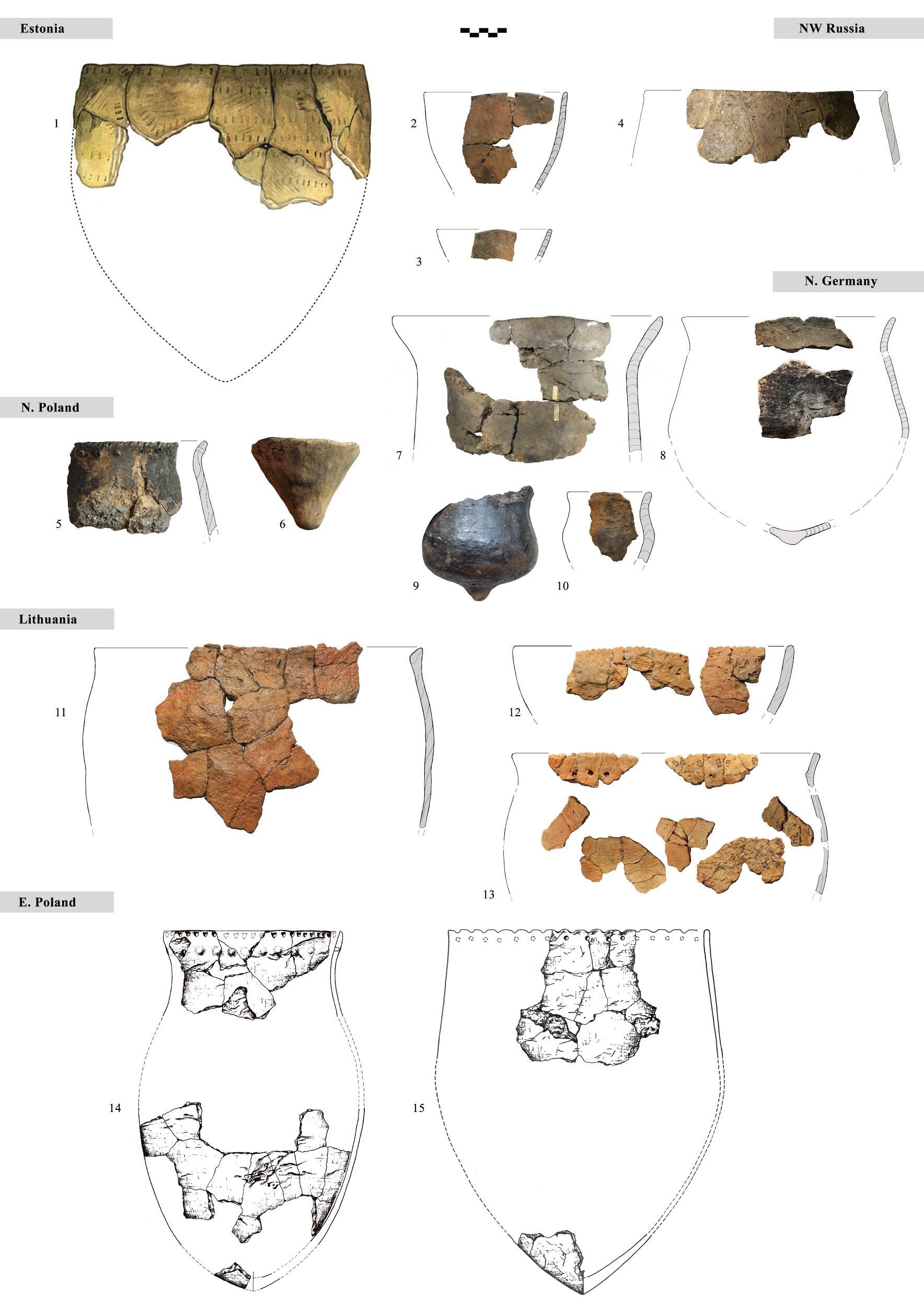


**Fig. S2.** Hunter-gatherer pottery from Estonia, North-west Russia, Northern and Eastern Poland, Northern Germany and Lithuania during the late 6^th^- early 4^th^ millennium cal BC. 1- Kääpa (after [[14]](https://paperpile.com/c/eHRMPd/Ecdba)); 2-3 - Narva Joaorg; 4 - Rudnya Serteyskaya; 5-6 - Dąbki, site 9; 7-10 - Grube-Rosenhof LA 58; 11 - Varėnė 10; 12 - Glūkas 3; 13 - Karaviškės 6; 14 - Krzemienne (after [[20]](https://paperpile.com/c/eHRMPd/xHGdf)); 15 - Bransk, site 22 (after [[21]](https://paperpile.com/c/eHRMPd/3Qtyb)). Scale: 5 cm.

**
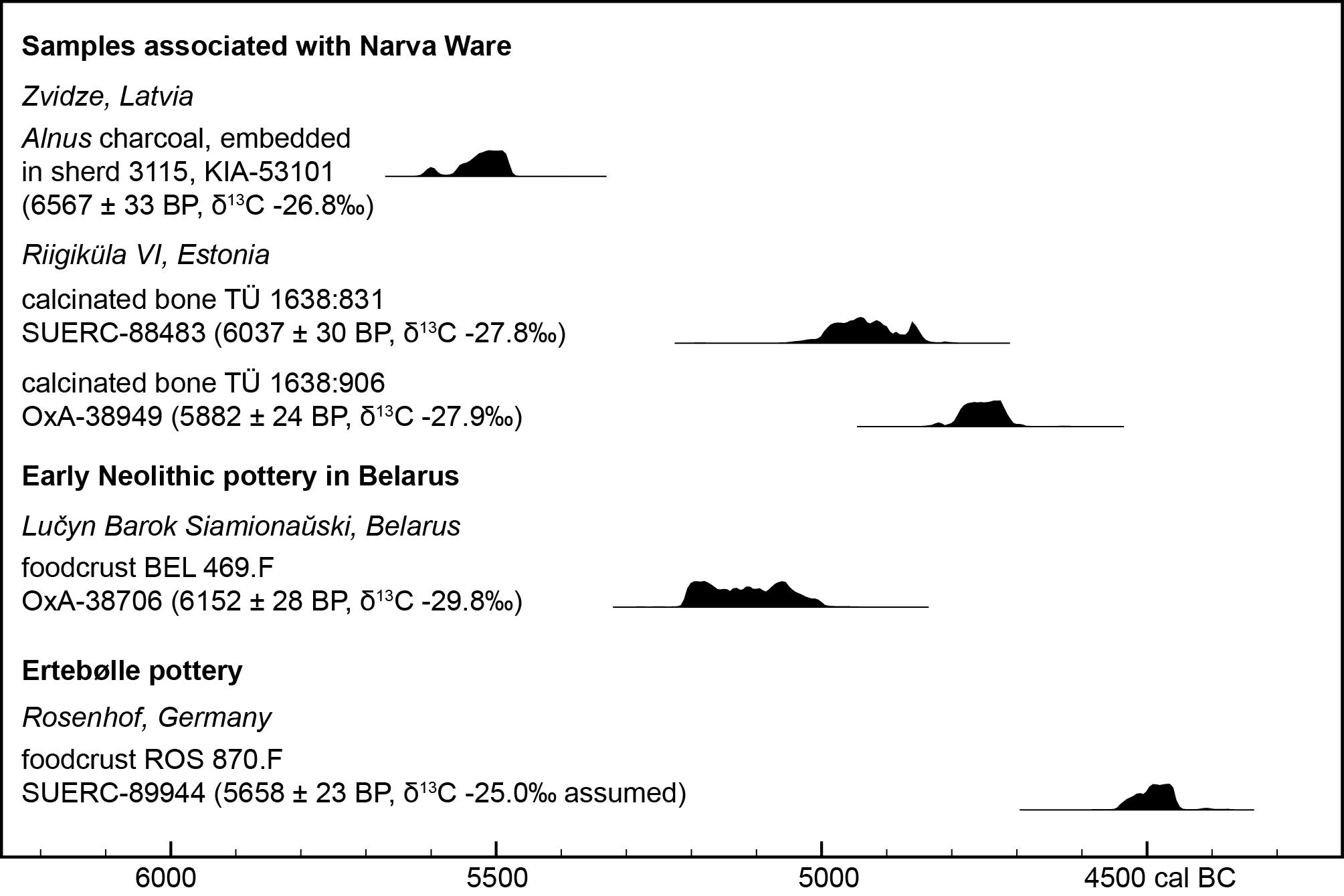
**

**Fig. S3.** New AMS radiocarbon (^14^C) dates associated with early pottery. Conventional ^14^C ages were calibrated using [[22]](https://paperpile.com/c/eHRMPd/6ZdDb) and [[23]](https://paperpile.com/c/eHRMPd/1ZlAr).


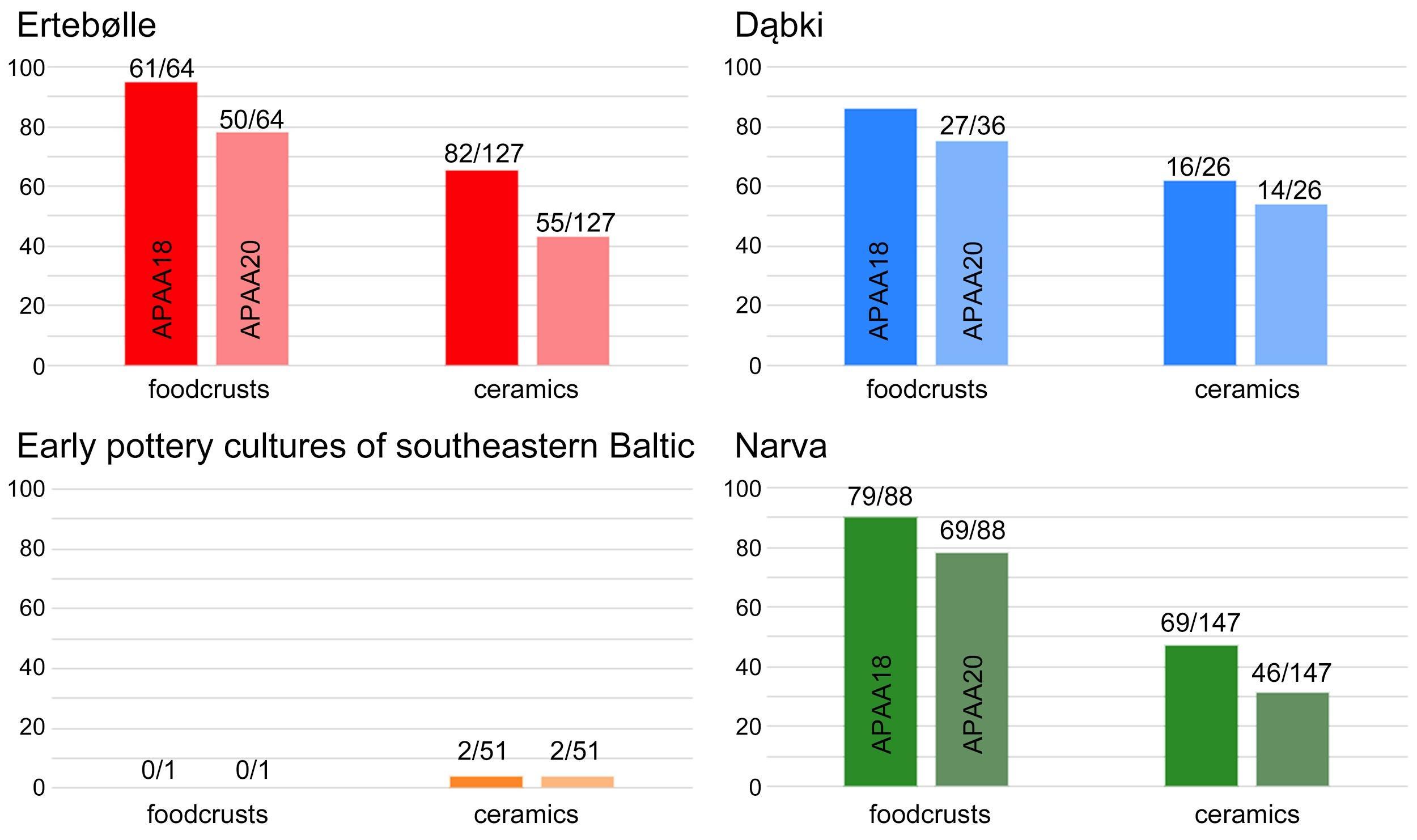


**Fig. S4** Proportion of foodcrusts and ceramic sherds (%) containing the *ω*-(*o*-alkylphenyl)alkanoic acids C_18_ and C_20_.


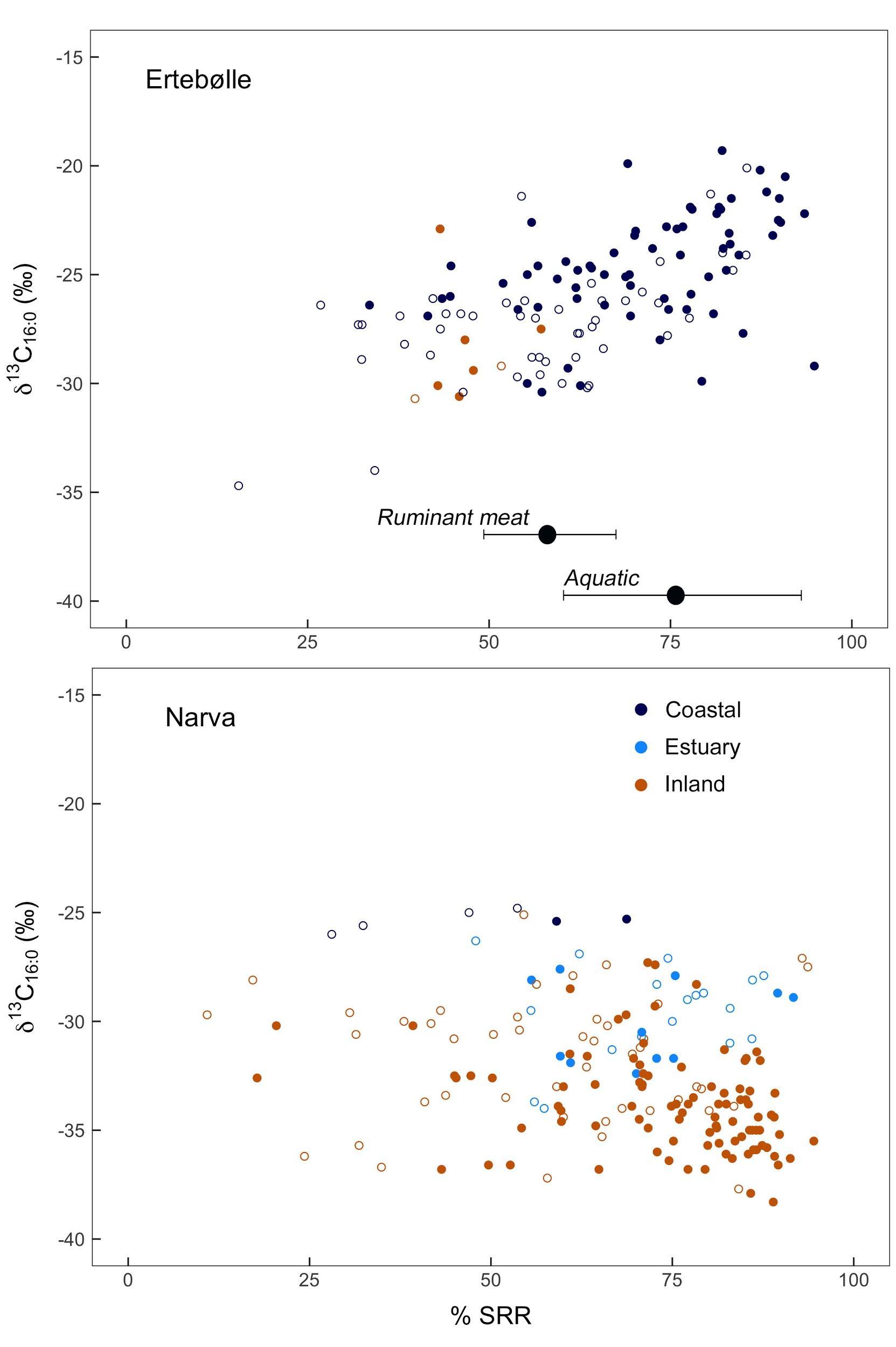


**Fig. S5**. δ^13^C values of palmitic acid (C_16:0_) against the *SRR*% (SRR/RRR) of phytanic acid obtained from pottery recovered from coastal, estuarine and inland sites dating to the Ertebølle and Narva cultures.


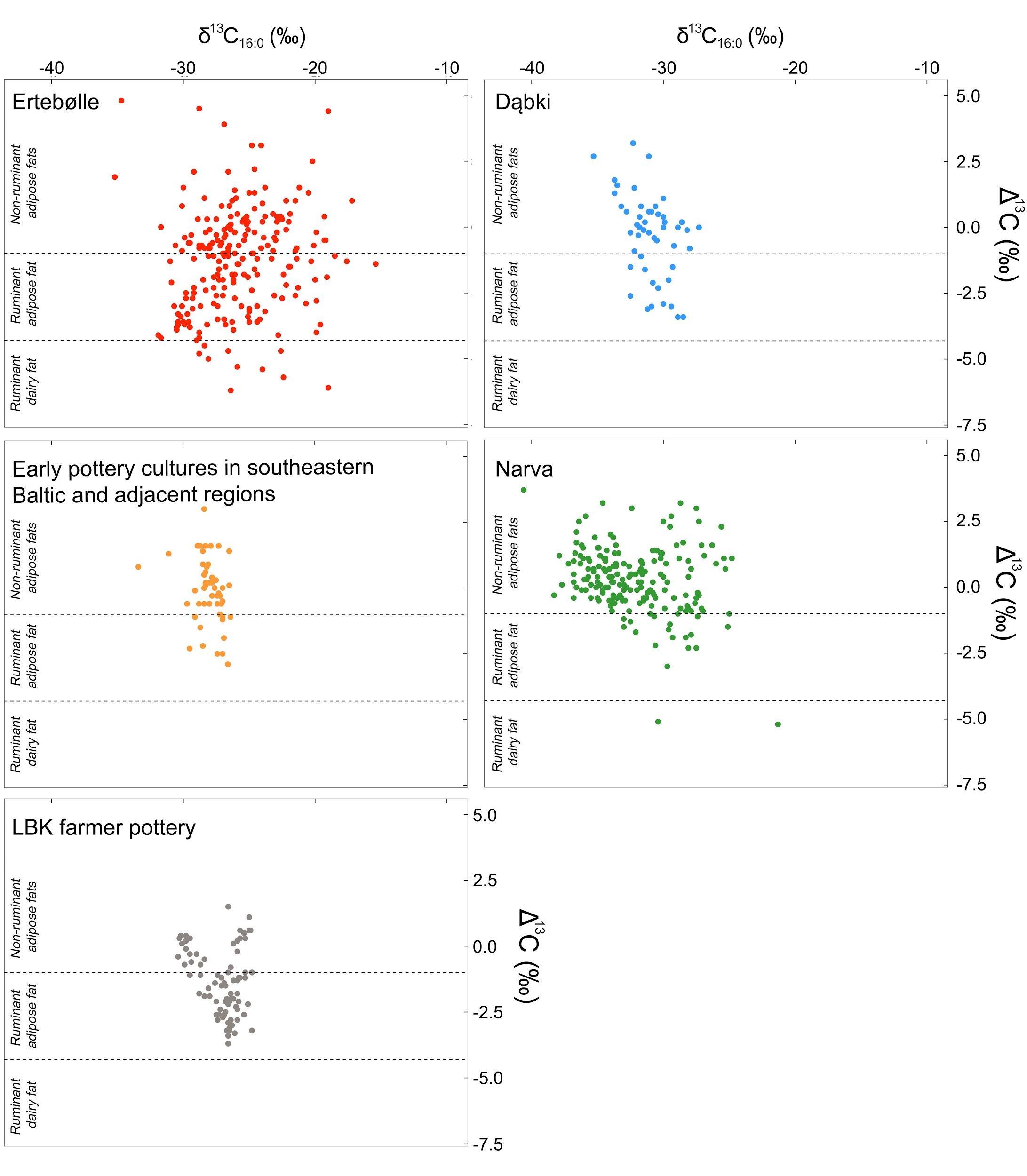


**Fig. S6**. Δ^13^C (δ^13^C_18:0_-δ^13^C_16:0_) against δ^13^C_16:0_ values obtained from Ertebølle, Dąbki Mesolithic, South-eastern Baltic and Narva cooking vessels throughout the Baltic Sea region. Values below -1.0 and -4.3‰, respectively, define a ruminant and/or dairy source of the lipids [[2,24]](https://paperpile.com/c/eHRMPd/DWftW+H7sBG). For comparison, cooking vessels of the LBK are plotted.

**
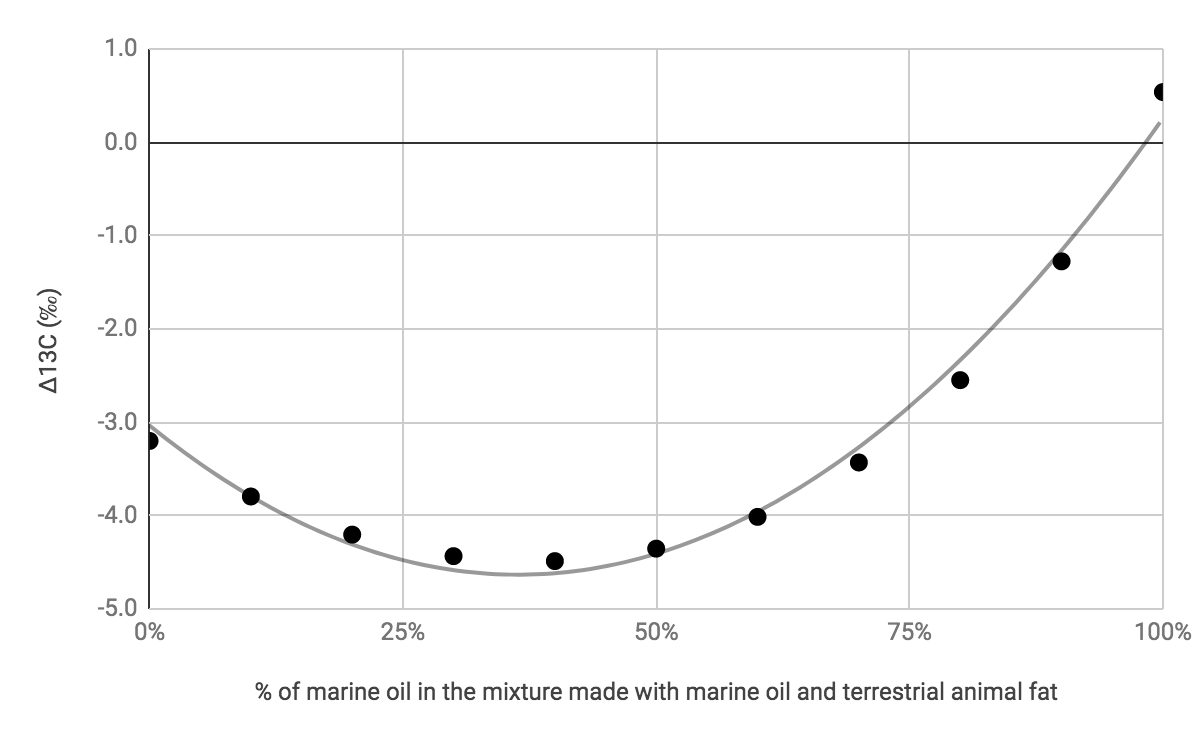
**

**Fig. S7.** Theoretical Δ^13^C (‰) mixing curve obtained when an increasing quantity of aquatic oil (δ^13^C_16:0_ = -22.7‰, δ^13^C_18:0_ = -22.1‰) is added to terrestrial animal fat (δ^13^C_16:0_ = -29.2‰, δ^13^C_18:0_ = -32.4‰).


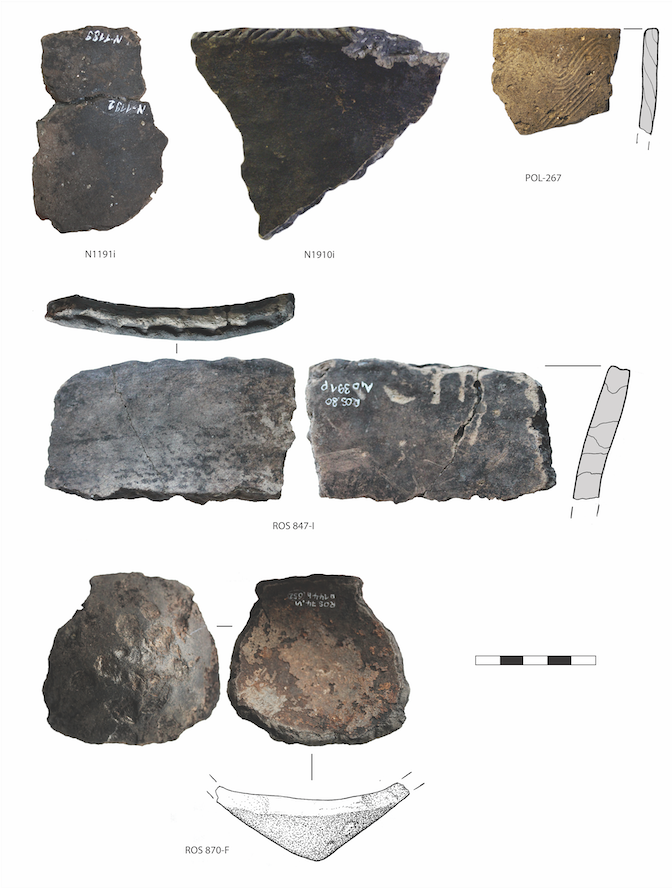


**Fig. S8.** The five vessels where dairy products are likely to have contributed to their δ^13^C values. Four of the vessels have been attributed to the Late Mesolithic Ertebølle culture (N1191i and N1910i from Neustadt and ROS 8476-I and ROS 870-F from Grube-Rosenhof), whilst the vessel from Kaldus (POL-267) is attributed to the Early Neolithic Narva culture. Scale: 5 cm.

**
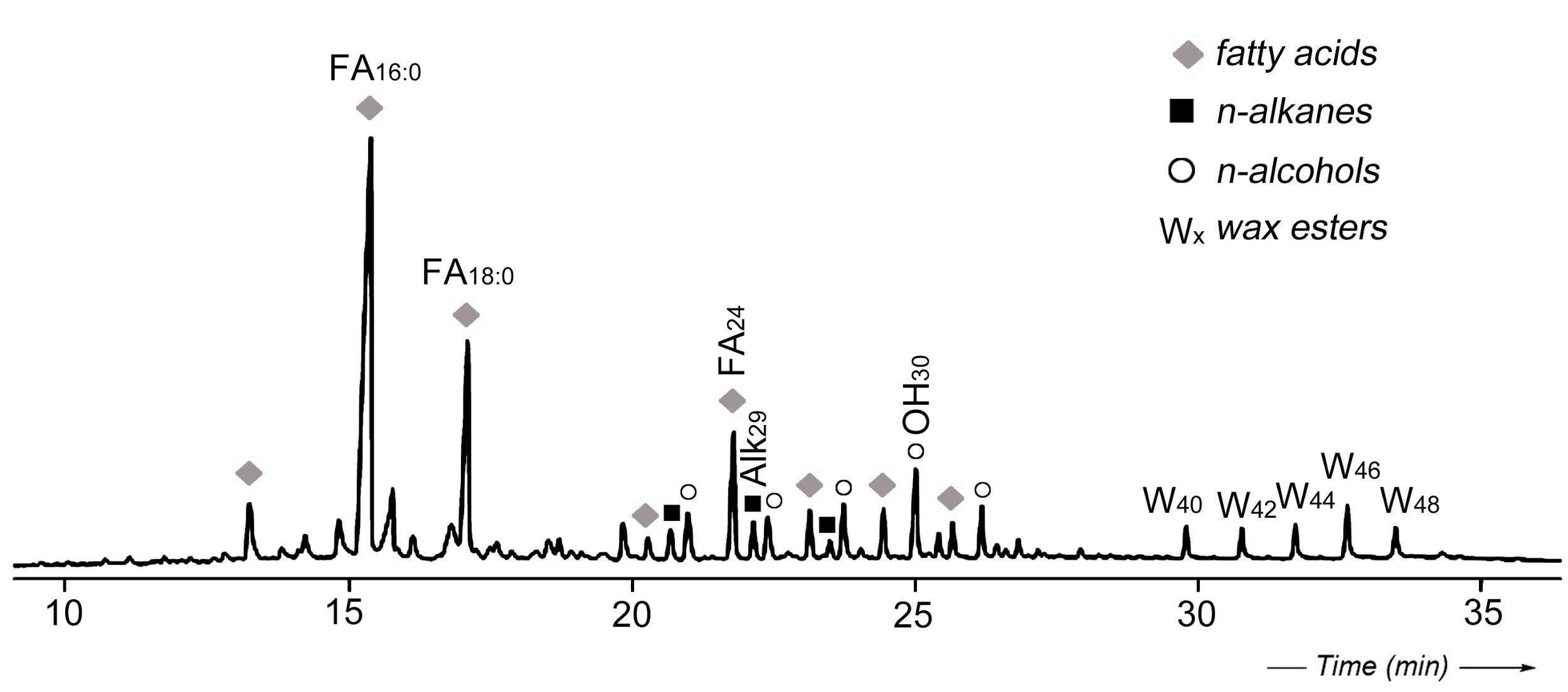
**

**Fig. S9.** Gas chromatogram of a solvent extract obtained from sample ROS 8.32-I (Grube-Rosenhof) showing the presence of beeswax. Fatty acids and alcohols were analysed as trimethylsilylated derivatives. N-alkanes, n-alkanols and palmitic acid wax esters meet the proposed criteria for beeswax as described by [[25]](https://paperpile.com/c/eHRMPd/PKYB).

**Supplementary Information: Tables**

**Table S1.** Archaeological sites in the Baltic area where cooking vessels belonging to hunter-gatherer cultures were sampled and for which molecular and isotopic data were used in the present research. The allocated number refers to the location on the map (Fig. S1).

| **#** | **Site** | **Nb. vessels** | **Nb. charred residues** | **Nb. potsherds** | **Samples with lipids*** | **GC-C-IRMS** | **References** |
| --- | --- | --- | --- | --- | --- | --- | --- |
| ***Ertebølle (n = 20 sites) - second half of 5^th^ millennium cal BC*** | | | | | | | |
| 1 | Åkonge | 5 | 3 | 5 | 8/8 | 8 | [[5,26]](https://paperpile.com/c/eHRMPd/G64D+4EYw) |
| 2 | Åle | 1 | - | 1 | 1/1 | 1 | [[26]](https://paperpile.com/c/eHRMPd/4EYw) |
| 3 | Bjørnsholm | 1 | - | 1 | 1/1 | 1 | [[26]](https://paperpile.com/c/eHRMPd/4EYw) |
| 4 | Flynderhage | 1 | 1 | - | 0/1 | - | This study |
| 5 | Frederiksodde | 1 | - | 1 | 1/1 | 1 | [[5]](https://paperpile.com/c/eHRMPd/G64D) |
| 6 | Gamborg Fjord | 1 | 1 | - | 1/1 | - | [[26]](https://paperpile.com/c/eHRMPd/4EYw); this study |
| 7 | Grube-Rosenhof LA 58 | 59 | 36 | 61 | 97/97 | 95 | This study |
| 8 | Havnø | 10 | - | 10 | 6/10 | 6 | [[26]](https://paperpile.com/c/eHRMPd/4EYw); this study |
| 9 | Hjarnø | 3 | 3 | - | 3/3 | 3 | This study |
| 10 | Kesemölla | 6 | - | 6 | 6/6 | 2 | [[27]](https://paperpile.com/c/eHRMPd/ZSkB) |
| 11 | Löddesborg | 24 | - | 24 | 24/24 | 8 | [[27]](https://paperpile.com/c/eHRMPd/ZSkB) |
| 12 | Neustadt LA 156 | 22 | 11 | 18 | 26/29 | 26 | [[5,26]](https://paperpile.com/c/eHRMPd/G64D+4EYw) ; this study |
| 13 | Ringkloster | 29 | 1 | 32 | 17/33 | 17 | [[26,28]](https://paperpile.com/c/eHRMPd/ECic+4EYw); this study |
| 14 | Ronæs Skov | 5 | 7 | - | 7/7 | 2 | [[26]](https://paperpile.com/c/eHRMPd/4EYw); this study |
| 15 | Soldattorpet | 19 | - | 19 | 19/19 | 6 | [[27]](https://paperpile.com/c/eHRMPd/ZSkB) |
| 16 | Stenø | 2 | - | 2 | 2/2 | 2 | [[26]](https://paperpile.com/c/eHRMPd/4EYw) |
| 17 | Syltholm | 10 | 1 | 9 | 10/10 | 10 | [[27]](https://paperpile.com/c/eHRMPd/ZSkB); this study |
| 18 | Tybrind Vig | 47 | 10 | 53 | 32/63 | 29 | [[5,26,28]](https://paperpile.com/c/eHRMPd/G64D+ECic+4EYw); this study |
| 19 | Vik | 1 | 0 | 1 | 1/1 | 1 | [[27]](https://paperpile.com/c/eHRMPd/ZSkB) |
| *Total Ertebølle* | | *247* | *74* | *243* | *262/317* | *218* |  |
| ***Dąbki Mesolithic - late 5th millennium cal BC*** | | | | | | | |
| 20 | Dąbki, site 9 | 60 | 37 | 26 | 49/63 | 49 | This study |
| ***South-eastern Baltic hunter-gatherer-fisher settlements with early pottery (n = 18 sites) - late 6^th^ - early 4^th^ millennium cal BC*** | | | | | | | |
| 21 | Bransk, site 2 | 1 | - | 1 | 1/1 | 1 | This study |
| 22 | Drazdy 12 | 2 | - | 2 | 2/2 | 2 | This study |
| 23 | Dubičiai 3 | 1 | - | 1 | 1/1 | 1 | This study |
| 24 | Dubovy Loh 5 | 1 | - | 1 | 1/1 | 1 | This study |
| 25 | Glūkas 3 | 1 | - | 1 | 1/1 | 1 | This study |
| 26 | Grądy-Woniecko, site 1 | 6 | - | 6 | 6/6 | 5 | This study |
| 27 | Gribaša 4 | 2 | - | 2 | 2/2 | 2 | This study |
| 28 | Jara 2 | 1 | - | 1 | 1/1 | 1 | This study |
| 29 | Jeroniki, site 2 | 3 | - | 3 | 2/3 | 1 | This study |
| 30 | Kamen’ 6 | 14 | - | 14 | 14/14 | 14 | This study |
| 31 | Karaviškės 6 | 2 | - | 2 | 2/2 | 2 | This study |
| 32 | Krzemienne, site 2 | 1 | - | 1 | 1/1 | 1 | This study |
| 33 | Lučyn Barok Siamionaŭski | 1 | 1 | 2 | 3/3 | 3 | This study |
| 34 | Rusakova | 2 | - | 2 | 2/2 | 2 | This study |
| 35 | Sien’čycy 3 | 1 | - | 1 | 1/1 | 1 | This study |
| 36 | Sośnia, site 1 | 4 | - | 4 | 4/4 | 4 | This study |
| 37 | Stacze, site 1 | 6 | - | 6 | 6/6 | 6 | This study |
| 38 | Varėnė 10 | 1 | - | 1 | 1/1 | 1 | This study |
| *Total South-eastern Baltic pottery cultures* | | *50* | *1* | *51* | *51/52* | *49* |  |
| ***Narva (n = 24 sites) - second half of 5^th^ millennium cal BC*** | | | | | | | |
| 39 | Akali | 6 | 2 | 6 | 8/8 | 5 | [[29]](https://paperpile.com/c/eHRMPd/4Cbs) |
| 40 | Asaviec 4 | 3 | - | 3 | 3/3 | 3 | This study |
| 41 | Biarešča 4 | 4 | - | 4 | 4/4 | 4 | This study |
| 42 | Daktariškė 5 | 5 | 5 | - | 5/5 | 5 | This study |
| 43 | Iča | 3 | 1 | 3 | 3/4 | 4 | This study |
| 44 | Kääpa | 12 | 12 | 10 | 22/22 | 13 | [[29]](https://paperpile.com/c/eHRMPd/4Cbs) |
| 45 | Kaldus, site 3 | 2 | - | 2 | 1/2 | 1 | This study |
| 46 | Kalmaküla | 2 | - | 2 | 1/2 | - | [[29]](https://paperpile.com/c/eHRMPd/4Cbs) |
| 47 | Kõnnu | 5 | - | 5 | 5/5 | 4 | [[29]](https://paperpile.com/c/eHRMPd/4Cbs) |
| 48 | Kõpu 1 | 1 | - | 1 | 1/1 | - | [[29]](https://paperpile.com/c/eHRMPd/4Cbs) |
| 49 | Kretuonas 1 | 8 | 1 | 8 | 9/9 | 9 | This study |
| 50 | Kroodi | 1 | 1 | 1 | 1/2 | 1 | [[29]](https://paperpile.com/c/eHRMPd/4Cbs) |
| 51 | Lommi III | 5 | - | 5 | 5/5 | 2 | [[29]](https://paperpile.com/c/eHRMPd/4Cbs) |
| 52 | Narva Joaorg | 34 | 12 | 34 | 41/46 | 27 | [[29]](https://paperpile.com/c/eHRMPd/4Cbs); this study |
| 53 | Osa | 24 | 17 | 17 | - | 34 | This study |
| 54 | Riigiküla IV, VI | 4 | - | 5 | 5/5 | - | [[29]](https://paperpile.com/c/eHRMPd/4Cbs) |
| 55 | Rudnya Serteyskaya** | 5 | 5 | - | 5/5 | 5 | This study |
| 56 | Ruhnu II | 1 | - | 1 | 1/1 | 1 | [[29]](https://paperpile.com/c/eHRMPd/4Cbs) |
| 57 | Sasieczno, site 4 | 1 | - | 1 | 1/1 | - | This study |
| 58 | Serteya X, XIV** | 6 | 6 | - | 6/6 | 6 | This study |
| 59 | Vihasoo III | 5 | - | 5 | 5/5 | 3 | [[29]](https://paperpile.com/c/eHRMPd/4Cbs) |
| 60 | Welcz Wielki, site 10A | 2 | - | 2 | 2/2 | - | This study |
| 61 | Zacennie | 1 | 1 | 1 | 2/2 | 2 | This study |
| 62 | Zvidze | 31 | 25 | 31 | - | 56 | This study |
| *Total Narva* | | *171* | *88* | *147* | *136/145* | *185* |  |
| **Total** | | 528 | 200 | 467 | 498/577 | 501 |  |

*when extracted.

**pottery belonging to the Rudnya tradition sharing the common features/attributes of Narva vessels.

**Table S2.** Fatty acid isotopic values and concentrations for the different lipid groups.

| **Source** | **δ^13^C_16:0_ (‰)** | **δ^13^C_18:0_ (‰)** | **C_16:0_ (%)** | **C_18:0_ (%)** |
| --- | --- | --- | --- | --- |
| *Western Baltic* |  |  |  |  |
| Freshwater | -32.8 ± 2.6 | -32.6 ± 2.6 | 15.0 ± 3.6 | 4.0 ± 1.6 |
| Marine | -22.7 ± 2.7 | -22.1 ± 2.6 | 14.5 ± 4.3 | 4.0 ± 2.2 |
| Porcine | -26.5 ± 1.2 | -25.6 ± 1.2 | 20.5 ± 2.5 | 10.9 ± 1.7 |
| Ruminant | -29.3 ± 1.5 | -32.1 ± 1.4 | 21.6 ± 3.1 | 13.2 ± 3.0 |
| Ruminant adipose* | -29.7 ± 1.4 | -31.9 ± 1.4 | 21.1 ± 3.1 | 13.4 ± 3.0 |
| Dairy* | -28.1 ± 1.2 | -32.9 ± 1.4 | 27.3 ± 3.5 | 11.0 ± 1.7 |
| *Eastern Baltic* |  |  |  |  |
| Freshwater | -32.8 ± 2.5 | -32.4 ± 2.3 | 15.0 ± 3.6 | 4.0 ± 1.6 |
| Marine | -24.3 ± 1.5 | -24.1 ± 1.9 | 14.5 ± 4.3 | 4.0 ± 2.2 |
| Porcine | -27.2 ± 0.5 | -26.2 ± 0.6 | 20.5 ± 2.5 | 10.9 ± 1.7 |
| Ruminant | -29.2 ± 1.9 | -32.3 ± 1.6 | 21.6 ± 3.1 | 13.2 ± 3.0 |
| Ruminant adipose* | -30.1 ± 1.9 | -31.8 ± 1.7 | 21.1 ± 3.1 | 13.4 ± 3.0 |
| Dairy* | -28.1 ± 1.2 | -32.9 ± 1.4 | 27.3 ± 3.5 | 11.0 ± 1.7 |

*only for the second model

**Table S3.** New δ^13^C values of palmitic and stearic acids obtained from modern animal tissues and used as reference, together with published data, to construct the ellipses in Fig. 2. δ^13^C values measured by GC-C-IRMS were corrected to take into account modern atmospheric δ^13^C values that have significantly decreased since the Industrial Revolution (Suess effect; [[9]](https://paperpile.com/c/eHRMPd/Sxa9i)).

| **Resource group** | **Common name** | **Binomial name** | **Provenience** | **Estimated year of death** | **δ^13^C_16:0_** | **δ^13^C_18:0_** |
| --- | --- | --- | --- | --- | --- | --- |
| Ruminant adipose fat | Cattle | *Bos taurus* | Estonia | 2016 | -28.0 | -30.1 |
| Ruminant adipose fat | Sheep | *Ovis aries* | Estonia | 2015 | -30.3 | -31.8 |
| Ruminant adipose fat | Eurasian elk | *Alces alces* | Estonia | 2015 | -30.0 | -32.0 |
| Ruminant adipose fat | Eurasian elk | *Alces alces* | Estonia | 2016 | -31.2 | -32.7 |
| Ruminant adipose fat | Eurasian elk | *Alces alces* | Estonia | 2016 | -31.3 | -33.0 |
| Ruminant adipose fat | Eurasian elk | *Alces alces* | Estonia | 2016 | -31.6 | -33.0 |
| Ruminant adipose fat | Eurasian elk | *Alces alces* | Estonia | 2016 | -33.4 | -34.7 |
| Ruminant adipose fat | Eurasian elk | *Alces alces* | Estonia | 2016 | -31.1 | -33.3 |
| Ruminant adipose fat | Eurasian elk | *Alces alces* | Estonia | 2016 | -30.1 | -30.7 |
| Ruminant adipose fat | Eurasian elk | *Alces alces* | Estonia | 2016 | -32.8 | -34.5 |
| Ruminant adipose fat | Eurasian elk | *Alces alces* | Estonia | 2016 | -29.4 | -31.4 |
| Ruminant adipose fat | Eurasian elk | *Alces alces* | Estonia | 2016 | -32.2 | -32.6 |
| Ruminant adipose fat | Eurasian elk | *Alces alces* | Estonia | 2016 | -30.8 | -32.1 |
| Ruminant adipose fat | Eurasian elk | *Alces alces* | Estonia | 2016 | -30.7 | -32.3 |
| Ruminant adipose fat | Eurasian elk | *Alces alces* | Estonia | 2016 | -32.6 | -33.9 |
| Porcine | Wild boar | *Sus scrofa* | Estonia | 2015 | -26.9 | -25.8 |
| Dairy fat | Cattle (milk) | *Bos taurus* | Estonia | 2015 | -25.8 | -34.5 |
| Freshwater | Roach | *Rutilus rutilus* | Estonia | 2015 | -29.6 | -29.6 |
| Freshwater | Tench | *Tinca tinca* | Estonia | 2017 | -32.7 | -31.1 |
| Freshwater | Perch | *Perca fluviatilis* | Estonia | 2017 | -31.8 | -31.0 |
| Freshwater | Perch | *Perca fluviatilis* | Estonia | 2017 | -33.9 | -31.6 |
| Freshwater | Pike | *Esox lucius* | Estonia | 2017 | -32.8 | -31.6 |
| Freshwater | Gudgeon | *Gobio gobio* | Estonia | 2017 | -36.2 | -35.4 |
| Freshwater | Gudgeon | *Gobio gobio* | Estonia | 2017 | -36.2 | -35.1 |
| Freshwater | Roach | *Rutilus rutilus* | Estonia | 2017 | -35.5 | -34.8 |
| Freshwater | Roach | *Rutilus rutilus* | Estonia | 2017 | -36.1 | -35.8 |
| Freshwater | Roach | *Rutilus rutilus* | Estonia | 2017 | -37.2 | -36.0 |
| Freshwater | Roach | *Rutilus rutilus* | Estonia | 2017 | -33.9 | -33.7 |
| Freshwater | Pike | *Esox lucius* | Estonia | 2017 | -32.6 | -31.7 |
| Freshwater | Pike | *Esox lucius* | Estonia | 2017 | -32.8 | -31.7 |
| Freshwater | Eurasian beaver | *Castor fiber* | Estonia | 2016 | -30.1 | -30.3 |
| Freshwater | Eurasian beaver | *Castor fiber* | Estonia | 2016 | -31.4 | -32.0 |
| Freshwater | Eurasian beaver | *Castor fiber* | Estonia | 2016 | -31.1 | -31.6 |
| Freshwater | Eurasian beaver | *Castor fiber* | Estonia | 2016 | -31.2 | -31.6 |
| Freshwater | Eurasian beaver | *Castor fiber* | Estonia | 2016 | -30.1 | -30.2 |
| Freshwater | Eurasian beaver | *Castor fiber* | Estonia | 2016 | -29.4 | -29.7 |
| Freshwater | Eurasian beaver | *Castor fiber* | Estonia | 2016 | -31.0 | -30.7 |
| Freshwater | Eurasian beaver | *Castor fiber* | Estonia | 2016 | -31.3 | -32.0 |
| Marine (Baltic Sea) | Baltic herring | *Clupea harengus membras* | Baltic Sea | 2015 | -23.7 | -23.2 |
| Marine (Baltic Sea) | European flounder | *Platichthys flesus* | Baltic Sea | 2015 | -24.3 | -24.4 |
| Marine (Baltic Sea) | Grey seal | *Halichoerus grypus* | Estonia | 2016 | -24.0 | -24.4 |
| Marine (Baltic Sea) | Pike | *Esox lucius* | Denmark | 2002 | -20.1 | -21.8 |

**Table S4.** Categorized NISP (Number of Identified Specimens) values for three of the ceramic-producing hunter-gatherer sites sampled in this study, Dąbki, Site 9 (Mesolithic) [[30,31]](https://paperpile.com/c/eHRMPd/i4kw+Tj8v), Grube-Rosenhof (Ertebølle culture) [[32]](https://paperpile.com/c/eHRMPd/GgRG) and Osa (Narva culture) [[19]](https://paperpile.com/c/eHRMPd/pmhzf). Note the overwhelming dominance of freshwater and marine fish in the assemblages, which are largely reflected in the use of pottery.

| **Category** | **Dąbki, Site 9** | **%** | **Grube-Rosenhof** | **%** | **Osa** | **%** |
| --- | --- | --- | --- | --- | --- | --- |
| Wild ruminants | 266 | 0.9 | 169 | 3.9 | 32 | 2.0 |
| Porcine | 238 | 0.8 | 56 | 1.3 | 90 | 5.7 |
| Other terrestrial mammals | 1437 | 5.0 | 63 | 1.5 | 56 | 3.6 |
| Marine mammals | 47 | 0.2 | 13 | 0.3 |  |  |
| Freshwater fish | 26298 | 92.2 | 365 | 8.5 | 1390 | 88.6 |
| Marine fish |  |  | 3278 | 76.2 |  |  |
| Migratory fish | 122 | 0.4 | 270 | 6.3 |  |  |
| Freshwater/marine fish |  |  | 37 | 0.9 |  |  |
| Birds | 60 | 0.2 | 46 | 1.1 |  |  |
| Amphibians and reptiles | 49 | 0.2 | 3 | 0.1 |  |  |
| **Total NISP** | 28517 | 100 | 4300 | 100 | 1568 | 100.0 |

**Table S5.** Samples where dairy products are likely to have made a significant contribution. The probability density distributions for dairy were obtained using a conservative mixing model.

| **Sample name** | **Sample ID*** | **Site** | **Cultural group** | **δ^13^C_16:0_** | **δ^13^C_18:0_** | **Δ^13^C** | **Mean (± SD)** | **2.5pc** | **Median** | **97.5pc** |
| --- | --- | --- | --- | --- | --- | --- | --- | --- | --- | --- |
| N1191i | B149 | Neustadt LA 156 | Ertebølle | -28.1 | -33.1 | -5.0 | 51% (±19%) | 8.3% | 53% | 82% |
| N1910i | B145 | Neustadt LA 156 | Ertebølle | -28.8 | -33.6 | -4.8 | 50% (±20%) | 7.1% | 52% | 81% |
| ROS 847-I | B426 | Grube-Rosenhof LA 58 | Ertebølle | -28.4 | -32.9 | -4.5 | 48% (±19%) | 7.1% | 50% | 80% |
| ROS 870-F | B487 | Grube-Rosenhof LA 58 | Ertebølle | -28.8 | -33.0 | -4.2 | 45% (±19%) | 5.4% | 46% | 78% |
| POL-267 | B653 | Kaldus, site 3 | Narva (imported ware) | -30.4 | -35.5 | -5.1 | 63% (±17%) | 18.8% | 65% | 89% |

*used in Dataset 1

**Table S6.** New δ^13^C values of palmitic and stearic acids obtained from modern non-ruminant animal tissues and used as reference (in addition to reference values published by [[8]](https://paperpile.com/c/eHRMPd/lVgUu)). δ^13^C values measured by GC-C-IRMS were corrected to take into account modern atmospheric δ^13^C values that have significantly decreased since the Industrial Revolution (Suess effect; [[9]](https://paperpile.com/c/eHRMPd/Sxa9i)).

| **Common name** | **Binomial name** | **Provenience** | **Estimated year of death** | **δ^13^C_16:0_** | **δ^13^C_18:0_** |
| --- | --- | --- | --- | --- | --- |
| Badger | *Meles meles* | Russia (Middle Don region) | 2018 | -27.4 | -24.1 |
| Beaver | *Castor fiber* | Estonia | 2016 | -31.4 | -32.1 |
| Beaver | *Castor fiber* | Estonia | 2016 | -31.0 | -31.6 |
| Beaver | *Castor fiber* | Estonia | 2016 | -31.2 | -31.7 |
| Beaver | *Castor fiber* | Estonia | 2016 | -30.0 | -30.2 |
| Beaver | *Castor fiber* | Estonia | 2016 | -29.3 | -29.7 |
| Beaver | *Castor fiber* | Estonia | 2016 | -31.0 | -30.8 |
| Beaver | *Castor fiber* | Estonia | 2016 | -31.3 | -32.1 |
| Beaver | *Castor fiber* | Russia (Middle Don region) | 2018 | -31.2 | -28.5 |
| Beaver | *Castor fiber* | Russia (Upper Volga region) | 2017 | -30.8 | -31.0 |
| Beaver | *Castor fiber* | Estonia | 2016 | -29.9 | -30.0 |
| Brown bear | *Ursus arctos* | Russia | 2018 | -28.9 | -29.1 |
| Brown bear | *Ursus arctos* | Estonia | 2015 | -26.6 | -27.3 |
| Fox | *Vulpes vulpes* | Russia (Middle Don region) | 2018 | -25.2 | -25.3 |
| Hare | *Lepus* sp. | Russia (Middle Don region) | 2018 | -30.8 | -30.3 |
| Marmot | *Marmota bobak* | Russia (Middle Don region) | 2018 | -31.7 | -29.6 |
| Marten | *Martes martes* | Russia (Middle Don region) | 2018 | -25.1 | -24.6 |
| Mink | *Mustela lutreola* | Russia (Middle Don region) | 2018 | -29.7 | -27.3 |
| Muskrat | *Ondatra zibethicus* | Russia (Middle Don region) | 2018 | -33.6 | -33.3 |
| Wild boar | *Sus scrofa* | Estonia | 2015 | -26.7 | -25.6 |
| Squirrel | Sciuridae | Russia (Middle Don region) | 2018 | -28.9 | -28.6 |

**References**

1. [Lucquin A, Colonese AC, Farrell TFG, Craig OE. 2016 Utilising phytanic acid diastereomers for the characterisation of archaeological lipid residues in pottery samples. *Tetrahedron Lett.* **57**, 703–707.](http://paperpile.com/b/eHRMPd/IHeKW)

2. [Craig OE, Allen RB, Thompson A, Stevens RE, Steele VJ, Heron CP. 2012 Distinguishing wild ruminant lipids by gas chromatography/combustion/ isotope ratio mass spectrometry. *Rapid Commun. Mass Spectrom.* **26**, 2359–2364.](http://paperpile.com/b/eHRMPd/H7sBG)

3. [Dudd SN. 1999 *Molecular and isotopic characterisation of animal fats in archaeological pottery*. Bristol, UK: University of Bristol.](http://paperpile.com/b/eHRMPd/tWjYf)

4. [Spangenberg JE, Jacomet S, Schibler J. 2006 Chemical analyses of organic residues in archaeological pottery from Arbon Bleiche 3, Switzerland - Evidence for dairying in the late Neolithic. *J. Archaeol. Sci.* **33**, 1–13.](http://paperpile.com/b/eHRMPd/GONNg)

5. [Craig OE *et al.* 2011 Ancient lipids reveal continuity in culinary practices across the transition to agriculture in Northern Europe. *Proceedings of the National Academy of Sciences* **108**, 17910–17915.](http://paperpile.com/b/eHRMPd/G64D)

6. [Spiteri CD. 2012 *Pottery use at the transition to agriculture in the western Mediterranean. Evidence from biomolecular and isotopic characterisation of organic residues in Impressed/Cardial Ware vessels*. York, UK: University of York.](http://paperpile.com/b/eHRMPd/qycmX)

7. [Cramp L *et al.* 2014 Neolithic dairy farming at the extreme of agriculture in northern Europe. *Proceedings of the Royal Society B: Biological Sciences* **281**, 20140819.](http://paperpile.com/b/eHRMPd/tuRk5)

8. [Pääkkönen M, Evershed RP, Asplund H. In press. Compound-specific stable carbon isotope values of modern terrestrial and aquatic animals from the Baltic Sea and Finland as an aid to interpretations of the origins of fatty acids preserved in archaeological pottery. *Journal of Nordic Archaeological Science*](http://paperpile.com/b/eHRMPd/lVgUu)

9. [Hellevang H, Aagaard P. 2015 Constraints on natural global atmospheric CO_2_ fluxes from 1860 to 2010 using a simplified explicit forward model. *Sci. Rep.* **5**, 17352.](http://paperpile.com/b/eHRMPd/Sxa9i)

10. [Deutsch B, Alling VKG, Humborg C, Korth F, Mörth CM. 2012 Tracing inputs of terrestrial high molecular weight dissolved organic matter within the Baltic Sea ecosystem.](http://paperpile.com/b/eHRMPd/nZ7xX) [*Biogeosciences* **9**, 4465–4475.](http://paperpile.com/b/eHRMPd/nZ7xX)

11. [Fernandes R, Millard AR, Brabec M, Nadeau MJ, Grootes P. 2014 Food reconstruction using isotopic transferred signals (FRUITS): A bayesian model for diet reconstruction. *PLoS ONE* **9**, 1–9.](http://paperpile.com/b/eHRMPd/bFjdh)

12. [Lucquin A *et al.* 2018 The impact of environmental change on the use of early pottery by East Asian hunter-gatherers. *Proceedings of the National Academy of Sciences* [**115**, 7931–7936](http://paperpile.com/b/eHRMPd/9zZd).](http://paperpile.com/b/eHRMPd/9zZd)

13. [Scheu A, Hartz S, Schmölcke U, Tresset A, Burger J, Bollongino R. 2008 Ancient DNA provides no evidence for independent domestication of cattle in Mesolithic Rosenhof, Northern Germany. *J. Archaeol. Sci.* **35**, 1257–1264.](http://paperpile.com/b/eHRMPd/M1WTp)

14. [Kriiska A, Oras E, Lõugas L, Meadows J, Lucquin A, Craig OE. 2017 Late Mesolithic Narva Stage in Estonia: Pottery, Settlement Types and Chronology. *Estonian Journal of Archaeology* **21**, 52-86.](http://paperpile.com/b/eHRMPd/Ecdba)

15. [Grootes PM, Nadeau M-J, Rieck A. 2004 ^14^C-AMS at the Leibniz-Labor: radiometric dating and isotope research. *Nucl. Instrum. Methods Phys. Res. B* **223-224**, 55–61.](http://paperpile.com/b/eHRMPd/vBNRk)

16. [Dunbar E, Cook GT, Naysmith P, Tripney BG, Xu S. 2016 AMS ^14^C Dating at the Scottish Universities Environmental Research Centre (SUERC) Radiocarbon Dating Laboratory – Corrigendum. *Radiocarbon* **58**, 233–233.](http://paperpile.com/b/eHRMPd/eRHgl)

17. [Brock F, Higham T, Ditchfield P, Ramsey CB. 2010 Current Pretreatment Methods for AMS Radiocarbon Dating at the Oxford Radiocarbon Accelerator Unit (Orau). *Radiocarbon* **52**, 103–112.](http://paperpile.com/b/eHRMPd/oStWo)

18. Kriiska A, Nordqvist K. 2012 Arheoloogilised väljakaevamised Narva-Jõesuu IIa neoliitilisel asulakohal 2010. aastal. *Narva Muuseumi toimetised* **12**, 14-37.

19. [Loze I. 1993 The Early Neolithic in Latvia. The Narva Culture. *Acta Archaeol.* **63**, 119–140.](http://paperpile.com/b/eHRMPd/pmhzf)

20. [Wawrusiewicz A. 2015 Ceramika wczesnosubneolityczna na podlasiu. Przyczynek do badań nad genezą kultury niemeńskiej. In *Супольнасці каменнага і бронзавага вякоў міжрзчча Віслыі Дняпра: Зборнік навуковых артыкулаў памяці Міхала Чарняўскага* (ed В.У. Ашэйчык, М.А. Плавінскі, В.М. Сідаровіч), pp. 123–142.](http://paperpile.com/b/eHRMPd/xHGdf)

21. [Wawrusiewicz A. 2013 Ceramika typu Sokołówek na Podlasiu i jej znaczenie w rozwoju społeczności subneolitycznych Polski północno-wschodniej. *Studia i materiały do badań nad neolitem i wczesną epoką brązu na mazowszu i podlasiu* **III**,](http://paperpile.com/b/eHRMPd/3Qtyb) 5-24*.*

22. [Ramsey CB. 2009 Bayesian Analysis of Radiocarbon Dates. *Radiocarbon* **51**, 337–360.](http://paperpile.com/b/eHRMPd/6ZdDb)

23. [Reimer PJ *et al.* 2013 IntCal13 and Marine13 Radiocarbon Age Calibration Curves 0–50,000 Years cal BP. *Radiocarbon* **55**, 1869–1887.](http://paperpile.com/b/eHRMPd/1ZlAr)

24. [Copley MS, Berstan R, Dudd SN, Docherty G, Mukherjee AJ, Straker V, Payne S, Evershed RP. 2003 Direct chemical evidence for widespread dairying in prehistoric Britain. *Proceedings of the National Academy of Sciences* **100**, 1524–1529.](http://paperpile.com/b/eHRMPd/DWftW)

25. [Roffett-Salque M *et al.* 2015 Widespread exploitation of the honeybee by early Neolithic farmers. *Nature* **527**, 226–230.](http://paperpile.com/b/eHRMPd/PKYB)

26. [Robson HK. 2015 *Evaluating the change of consumption and culinary practices at the transition to agriculture: a multi-disciplinary approach from a Danish kitchen midden*. York, UK: University of York.](http://paperpile.com/b/eHRMPd/4EYw)

27. [Papakosta V, Oras E, Isaksson S. 2019 Early pottery use across the Baltic – A comparative lipid residue study on Ertebølle and Narva ceramics from coastal hunter-gatherer sites in southern Scandinavia, northern Germany and Estonia. *Journal of Archaeological Science: Reports* **24**, 142–151.](http://paperpile.com/b/eHRMPd/ZSkB)

28. [Craig OE, Forster M, Andersen SH, Koch E, Crombé P, Milner NJ, Stern B, Bailey GN, Heron CP. 2007 Molecular and isotopic demonstration of the processing of aquatic products in northern European prehistoric pottery. *Archaeometry* **49**, 135–152.](http://paperpile.com/b/eHRMPd/ECic)

29. [Oras E, Lucquin A, Lõugas L, Tõrv M, Kriiska A, Craig OE. 2017 The adoption of pottery by north-east European hunter-gatherers: Evidence from lipid residue analysis. *J. Archaeol. Sci.* **78**, 112–119.](http://paperpile.com/b/eHRMPd/4Cbs)

30. [Schmölcke U, Nikulina E. 2015 Mesolithic beaver hunting station or base camp of supraregional Stone Age fur trade ? New archaeozoological and archaeogenetic results from Dąbki 9. In *The Dąbki site in Pomerania and the Neolithisation of the North European Lowlands (c. 5000-3000 calBC)*](http://paperpile.com/b/eHRMPd/i4kw) (eds J Kabaciński, S Hartz, DCM Raemaekers, T Terberger)[, pp. 65–86](http://paperpile.com/b/eHRMPd/i4kw). Leidorf, Germany: Archäologie und Geschichte im Ostseeraum, Band 8. Rahden/Westfälische.

31. [Zabilska-Kunek M, Makowiecki D, Robson HK. 2015 New archaeoichthyological data from the settlement at Dąbki. In *The Dabki site in Pomerania and the Neolithisation of the North European Lowlands (c. 5000-3000 calBC)*](http://paperpile.com/b/eHRMPd/Tj8v) (eds J Kabaciński, S Hartz, DCM Raemaekers, T Terberger)[, pp. 87–112.](http://paperpile.com/b/eHRMPd/Tj8v) Leidorf, Germany: Archäologie und Geschichte im Ostseeraum, Band 8. Rahden/Westfälische.

32. [Hartz S, Jöns H, Lübke H, Schmölcke U, Carnap-Bornheim (von) C, Heinrich D, Klooss S, Lüth F, Wolters S. 2014 Prehistoric settlements in the south-western Baltic sea and development of the regional Stone Age economy. Final report of the SINCOS-II-subproject 4.](http://paperpile.com/b/eHRMPd/GgRG)  *Bericht der Römisch-Germanischen Kommission* **92**, 77–210.
